# Homoleptic octahedral Co^II^ complexes as precatalysts for regioselective hydroboration of alkenes with high turnover frequencies†

**DOI:** 10.1039/d3ra06113b

**Published:** 2023-09-22

**Authors:** Guoqi Zhang, Haisu Zeng, Nora Zadori, Camila Marino, Shengping Zheng, Michelle C. Neary

**Affiliations:** a Department of Sciences, John Jay College and PhD Program in Chemistry, The Graduate Center of the City University of New York New York 10019 NY USA guzhang@jjay.cuny.edu; b Department of Chemistry, Hunter College, The City University of New York New York 10065 NY USA

## Abstract

Homoleptic complexes adopting octahedral coordination modes are usually less active in catalysis due to the saturated coordination around metal centers that prevents substrate activation in a catalytic event. In this work, we demonstrated that a homoleptic octahedral cobalt complex (1) of 4′-pyridyl-2,2′;6′,2′′-terpyridine that experienced monoprotonation at the non-coordinating pyridyl moiety upon crystallization could serve as a highly efficient precatalyst for the hydroboration of styrene derivatives with Markovnikov selectivity. The solid-state structure of this precatalyst along with relevant homoleptic Co^II^ and Fe^II^ complexes has been characterized by X-ray crystallography. In the solid state, 1 features one-dimensional hydrogen-bonded chains that are further stacked by interchain π⋯π interactions. The newly synthesized complexes (1–3) along with several known analogues (4–6) were examined as precatalysts for the hydroboration of alkenes. The best-performing system, 1/KO^*t*^Bu was found to promote Markovnikov hydroboration of substituted styrenes with high turnover frequencies (TOFs) up to ∼47 000 h^−1^, comparable to the most efficient polymeric catalyst [Co(pytpy)Cl_2_]_*n*_ reported to date. Although some limitations in substrate scope as well as functional group tolerance exist, the catalyst shows good promise for several relevant hydrofunctionaliation reactions.

## Abstract 2

Monoprotonated homoleptic complex of cobalt(ii) with 4′-pyridyl-2,2′;6′,2′′-terpyridine ligand is an efficient precatalyst for hydroboration of styrene derivatives with Markovnikov selectivity, displaying turnover frequencies up to 47 000 h^−1^.

## Graphical abstract

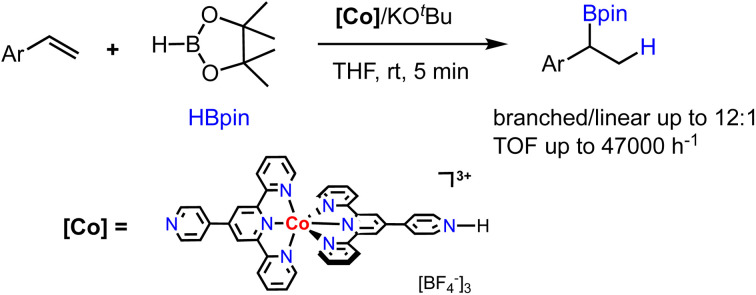

## Introduction

Hydrofunctionalization of unsaturated bonds provides a powerful tool for incorporating valuable functional groups into hydrocarbon compounds.^1^ In particular, alkene hydroboration is one of the most popular and convenient ways to approach alkylboronates that have extensive applications in C–C bond forming processes through cross-coupling reactions.^2^ Great advances have been made in the past decade toward developing metal-based catalysts utilizing various transition metals from precious Ru, Rh and Ir to earth abundant ones such as Fe, Co, Ni, Cu, and Mn,^3–7^ with the latter being the focus of recent research as nowadays chemists are seeking lower cost and more environmentally sustainable catalytic methodologies.

**Scheme 1:**
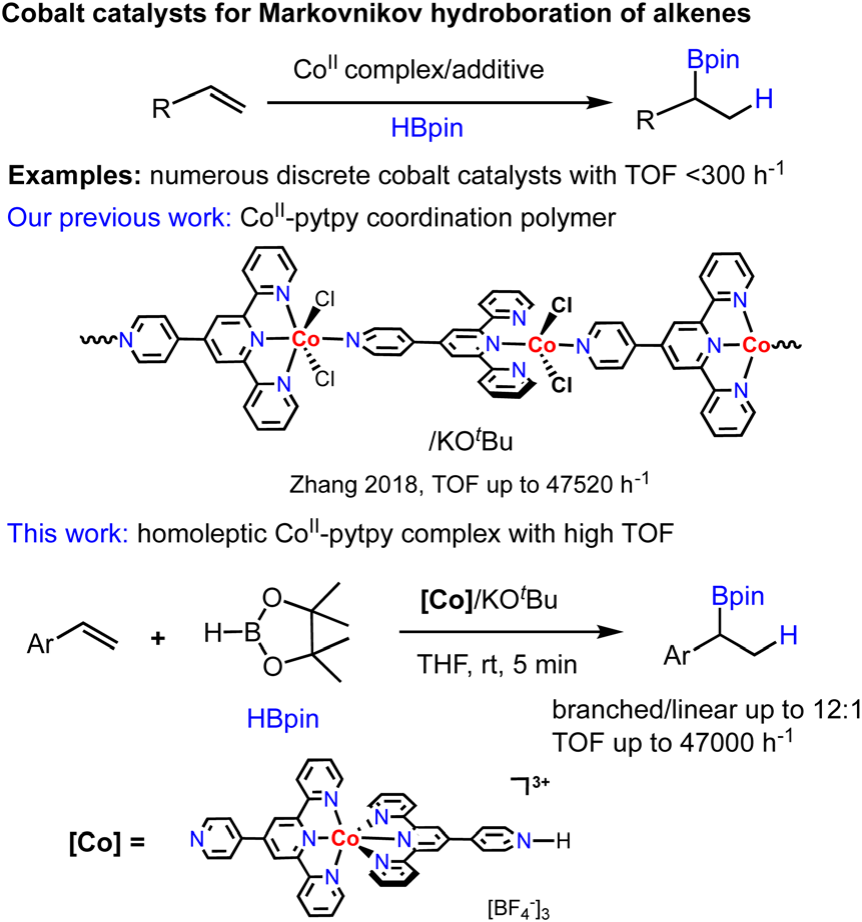
The development of Co-catalysed hydroboration of alkenes.

Among many well-defined earth-abundant metal catalysts for alkene hydroboration that have emerged over the past decade, cobalt catalysts turned out to be most attractive with respect to the ligand versatility, good regioselectivity of products and high TOFs.^8–13^ Numerous cobalt catalyst systems have been reported for either branched (Markovnikov) or linear (anti-Markovnikov) selectivity by Chirik,^9^ Lu,^10^ Thomas,^11^ Huang,^12^ Findlater^13^ and our group,^8^ respectively. While all cobalt-based discrete molecular catalysts reported thus far realized alkene hydroboration with relatively low TOFs (<300 h^−1^), we have disclosed that a one-dimensional Co^II^-coordination polymer assembled from a divergent ligand, 4′-pyridyl-2,2′; 6′,2′′-terpyridine (pytpy), enabled Markovnikov-selective hydroboration of aryl alkenes with high TOFs up to 47 520 h^−1^.^8*a*^ However, the origin of the extremely high catalytic efficiency of this polymeric cobalt catalyst remained unclear. To better understand whether the polymeric structure of catalyst is vital for the unexpected activity, we decided to explore discrete molecular analogues of the polymeric precatalyst by using the same pytpy ligand and different cobalt salts (Scheme 1).

**Scheme 2:**
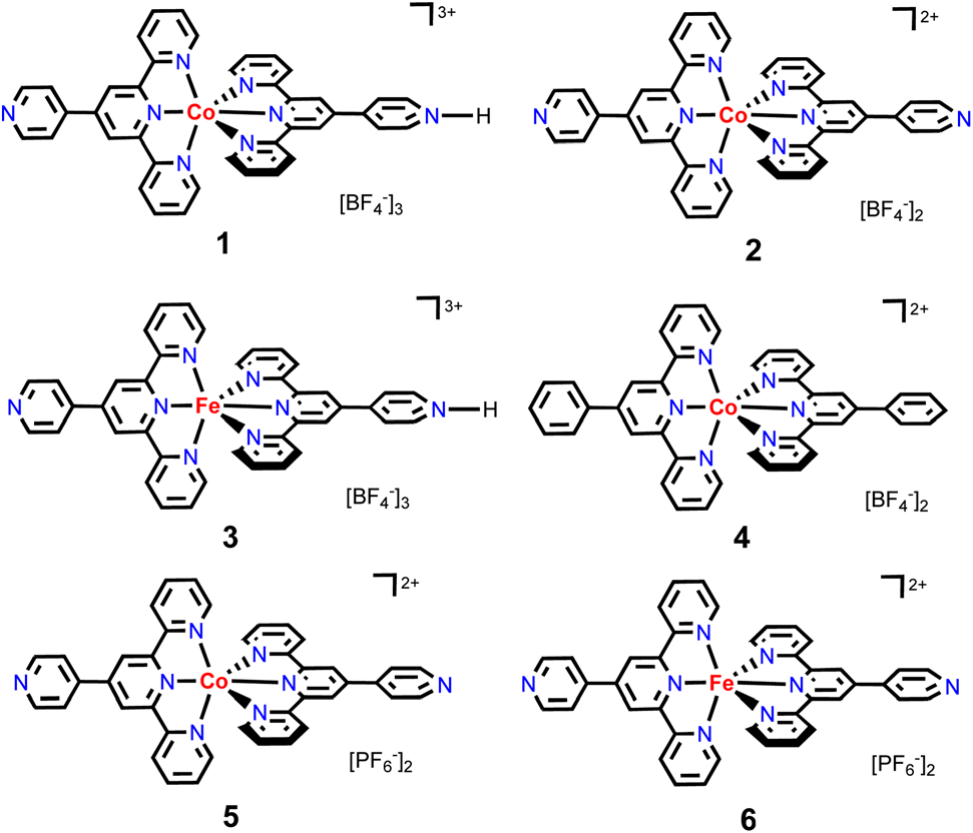
The structures of cobalt and iron complexes 1–6 studied in this work.

To continue our recent efforts on earth-abundant metal catalysis with tpy ligands,^14^ herein, we report the synthesis and structural characterization of homoleptic octahedral Co^II^ and Fe^II^ complexes of pytpy containing tetrafluoroborate or hexafluorophosphate counterions (Scheme 2), and their surprisingly high catalytic activity for regioselective hydroboration of alkenes with high TOFs. Although homoleptic octahedral metal complexes of tpy derivatives have been well explored for electrochemical and photophysical properties, and some have been utilized as supramolecular synthons, they are considered to be less catalytically active as the coordination environment of the metal centers makes it relatively inaccessible during a catalytic event. Nevertheless, several examples of homoleptic Co, Fe and Ni tpy complexes have been reported to promote electrocatalytic reduction of CO_2_,^15^ owing to their rich redox chemistry that can be tuned by varying electronic substituents on the tpy backbone.^16^

## Results and discussion

Carefully layering a solution of Co(BF_4_)_2_·6H_2_O in MeOH onto a CH_2_Cl_2_–MeOH solution (10 mL, 3 : 1, v/v) of pytpy over two weeks led to the formation of red block-like crystals of 1 that were suitable for single-crystal X-ray diffraction analysis. A bulk sample of the crystals has been isolated in 92% yield. The solution ^1^H NMR spectrum of 1 reveals broadened paramagnetic signals that could not be unambiguously assigned. The mass spectrum shows the only peak envelope at 679.1766 that can be assigned to the cation of the complex and the isotope pattern matches with that simulated. The solid-state structure of 1 was confirmed unambiguously by X-ray crystallography as a mono-protonated complex of [Co(pytpy)(H-pytpy)][BF_4_]_3_, a product obtained serendipitously during attempts to produce the expected complex [Co(pytpy)_2_][BF_4_]_2_. 1 crystallizes in the monoclinic space group *P*2_1_/*c*. The spontaneous partial protonation of pytpy ligand during the complexation with transition metals is reminiscent of known Ru and Fe complexes of the same ligand.^17,18^ The synthesis of 1 was well reproducible as evidenced from several independent layering experiments as well as the direct reaction between the ligand and Co(BF_4_)_2_·6H_2_O in a CH_2_Cl_2_–MeOH solution. In contrast, the originally expected homoleptic complex [Co(pytpy)_2_][BF_4_]_2_ (2) has been synthesized by adopting the standard reaction sequence, *i.e.* the solution reaction of pytpy and CoCl_2_·6H_2_O followed by an anion exchange with excess amount of NaBF_4_ (see ESI†). X-ray quality single crystals of 2 were obtained by vapor diffusion of diethyl ether into a solution of 2 in acetonitrile over 3 days. The mass spectrum of 2 shows the same peak envelope at 679.1766 as observed in 1. X-ray structural analysis confirmed the structure of 2 as expected and it crystallizes in the monoclinic space group *Pc*. Co-crystallized solvent molecules of CH_2_Cl_2_ and CH_3_CN were found in each cell of 1 and 2, respectively.

**Fig. 1:**
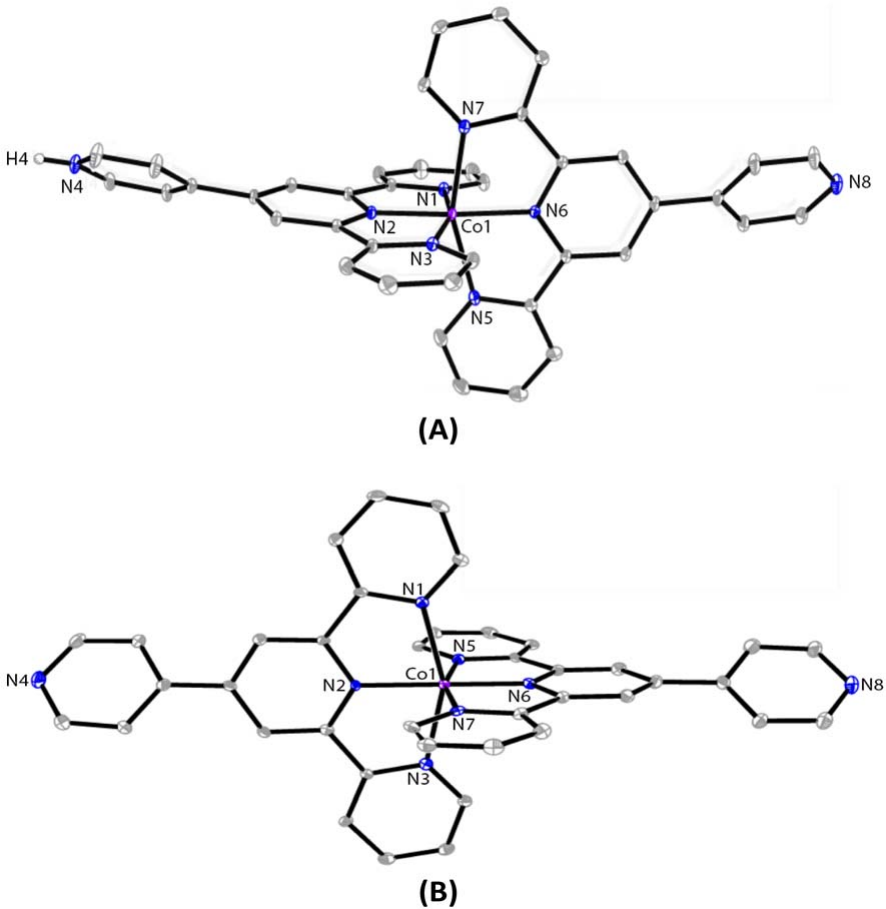
The ORTEP structures of 1 (A) and 2 (B) with thermal ellipsoids drawn at the 30% probability level. BF_4_^−^ counterions and H atoms bound to C are omitted for clarity. Selected bond parameters for 1: Co1–N1 = 1.998(2), Co1–N2 = 1.8675(17), Co1–N3 = 1.992(2), Co1–N5 = 2.150(2), Co1–N6 = 1.9270(18), Co1–N7 = 2.1519(19), N4–H4 = 0.84(9)Å, N1–Co1–N2 = 81.20(7), N2–Co1–N3 = 81.24(8), N5–Co1–N6 = 78.47(7), N6–Co1–N7 = 79.16(7)°; for 2: Co1–N1 = 2.154(4), Co1–N2 = 1.943(4), Co1–N3 = 2.182(4), Co1–N5 = 1.989(4), Co1–N6 = 1.875(4), Co1–N7 = 1.987(4)Å, N1–Co1–N2 = 78.62(16), N2–Co1–N3 = 77.99(16), N5–Co1–N6 = 81.33(16), N6–Co1–N7 = 80.78(17)°.

**Fig. 2:**
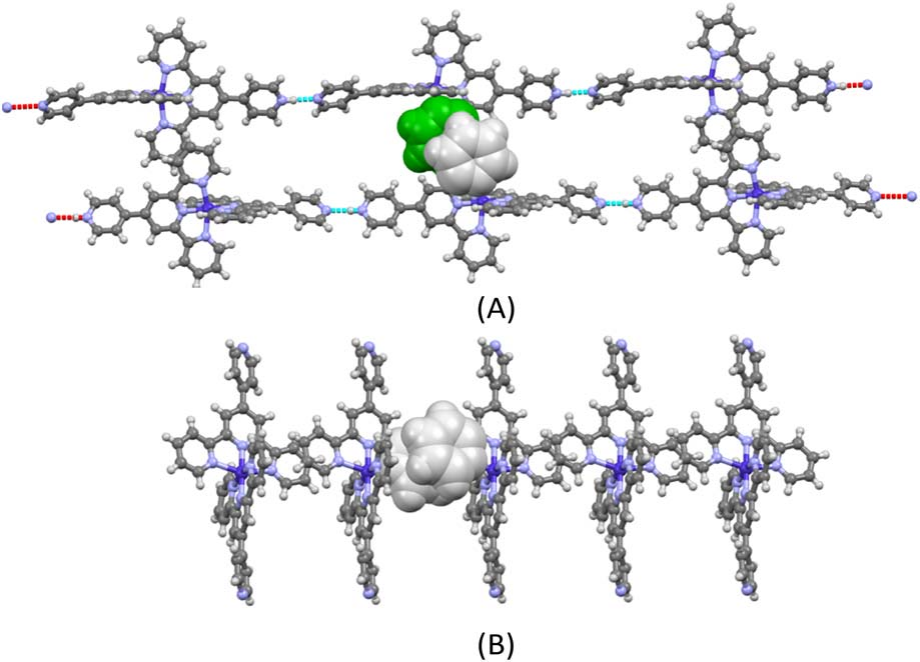
The 1-D hydrogen-bonded chains found in 1 and the π-stacking between the chains (A) and the intermolecular π-stacking in 2 (B). BF_4_^−^ counterions and co-crystallized solvents are omitted for clarity.

The ORTEP representations of cations of 1 and 2 are shown in Fig. 1, respectively. In the cations of 1 and 2, Co–N bond lengths around the cobalt coordination centers are within 1.8675(17)–2.1519(19) Å for 1 and 1.875(4)–2.182(4) Å for 2 (see caption of Fig. 1), which are unexceptional compared to the known crystal structures of Co(pytpy)_2_(PF_6_)_2_ complexes.^19^ The ligand conformations in 1 and 2 are not the same. In both structures, the non-coordinated pyridine ring is twisted with respect to the tpy domain to which it is attached. For 1, the angles between the least squares planes of the rings containing atoms N2 and N4, and N6 and N8 are 33.32° and 34.84°, respectively. For 2, the relevant twist angles are 40.36° and 34.71°. In addition, the deviation away from linearity of the N4⋯Co⋯N8 angle is notable for the cation of 1. The angle of N4⋯Co1⋯N8 is 167.73(2)° for the cation of 1, but it is closer to linear in the cation of 2 (177.44(5)°). This is likely due to the ligand protonation in 1 and the resulting formation of one-dimensional (1-D) hydrogen-bonded chains as well as the major interchain π⋯π stacking between ‘side-arm’ pyridine rings observed in 1 (Fig. 2). A similar situation has been reported in relevant Fe and Ru complexes.^17,18^ As seen in Fig. 2, the 1-D chain is assembled through N4–H4⋯N8(*i*) hydrogen bonds (symmetry code *i* = *x* − 1, *y*, *z* − 1, N4–H4 = 0.84(9) Å, H4⋯N8 = 1.82(9) Å, N4–H4⋯N8(*i*) = 172(7)°) and the chains are packed by π⋯π interaction of ‘side-arm’ pyridine rings (the closest C⋯C contact is 3.446(4) Å). Other types of π-stacking patterns are also observed in the 3-D packing framework of 1. The intermolecular packing in the cation of 2 is dominated by π⋯π stacking between ‘side-arm’ pyridine rings (the closest C⋯C contact is 3.604(7) Å), similar to that of 1.

**Fig. 3:**
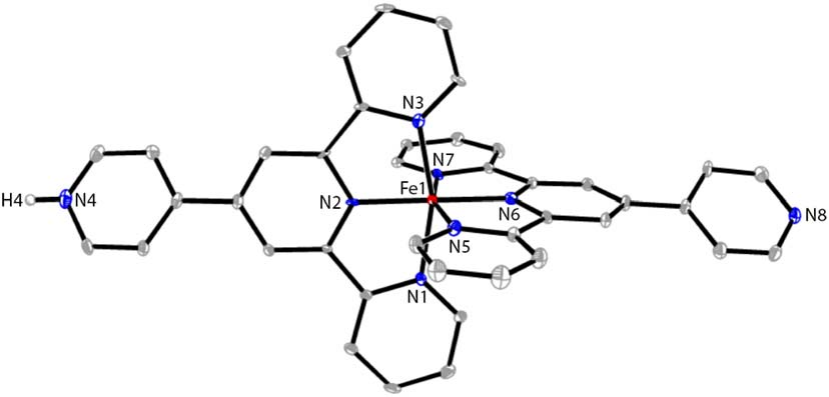
The ORTEP structure of 3 with thermal ellipsoids drawn at the 30% probability level. BF_4_^−^ counterions and H atoms bound to C are omitted for clarity. Selected bond parameters: Fe1–N1 = 1.982(5), Fe1–N2 = 1.882(5), Fe1–N3 = 1.972(5), Fe1–N5 = 1.972(5), Fe1–N6 = 1.876(5), Fe1–N7 = 1.976(5), N4–H4 = 0.88(2)Å, N1–Fe1–N2 = 81.0(2), N2–Fe1–N3 = 81.2(2), N5–Fe1–N6 = 80.6(2), N6–Fe1–N7 = 81.2(2)°.

Next, the reaction of pytpy with Fe(BF_4_)_2_·6H_2_O using the same layering method as for 1 resulted in the isolation of X-ray quality crystals of 3. Complex 3 is isomorphic to 1 and also crystallizes in the monoclinic space group *P*2_1_/*c*. A disordered solvent molecule in the asymmetric unit of 3 could not be well modelled and so it was treated as a diffuse contribution using PLATON/SQUEEZE.^20^ The monoprotonated cation of 3 is shown in Fig. 3, which has been reported previously in compounds [Fe(pytpy)(pytpyH)][Fe(NCS)_6_]·2H_2_O and [Fe(pytpy)(pytpyH)][Fe(NCS)_6_]·MeCN.^18^ The bending conformation in cation of 3 is very close to that found in 1 (angle N4⋯Fe1⋯N8 is 168.04(6)°), resulting from the intermolecular hydrogen bond N4–H4⋯N8(*i*) (symmetry code *i* = *x* + 1, *y*, *z* + 1, N4–H4 = 0.88(2) Å, H4⋯N8 = 1.80(3) Å, N4–H4⋯N8(*i*) = 167(8)°). Again, a similar 1-D hydrogen-bonded chain was found in 3. As referenced, homoleptic complexes 4–6 (Scheme 2) were synthesized according to the procedure reported previously.^21^

In order to evaluate whether new octahedral Co^II^ and Fe^II^ complexes could be used as effective precatalysts for alkene hydroboration, we first adopted the optimized conditions as for [Co(pytpy)Cl_2_]_*n*_ to perform catalytic hydroboration of styrene with pinacolborane (HBpin). The results of catalytic screening are summarized in Table 1. To our delight, when 1 (0.025 mol%) and KO^*t*^Bu (1 mol%) were combined in THF, the hydroboration of styrene was realized in 5 min to give the branched (7) and linear alkylboronates (8) in 98% total yield (TOF = ∼47 000 h^−1^ for both regioisomers) and 9 : 1 regioselectivity (entry 1, Table 1), closely comparable to the results obtained by using polymeric [Co(pytpy)Cl_2_]_*n*_ as a precatalyst. This is remarkable and represents the first homoleptic octahedral cobalt(ii) complex to enable alkene hydroboration with extremely high efficiency. Likewise, when 2 was used for the reaction under the same conditions, similar regioselectivity was found, while the yield dropped slightly to 80% (TOF = ∼38 000 h^−1^). Interestingly, going from Co^II^ to Fe^II^ resulted in a notable loss of catalytic activity, as 3 catalysed the reaction with 17% yield in 5 min (8000 h^−1^), although the regioselectivity remained (entry 3). In addition, both complexes 4 and 5 are moderately active precatalysts for styrene hydroboration (entries 4 and 5), indicating the importance of both the pytpy ligand and BF_4_^−^ counterions for high catalytic efficiency. According to these results, it is not surprising to see that complex 6, Fe(pytpy)_2_(PF_6_)_2_, is an inactive precatalyst (entry 6) under standard conditions.

**Table 1:** Condition screening for hydroboration of styrene with HBpin^a^

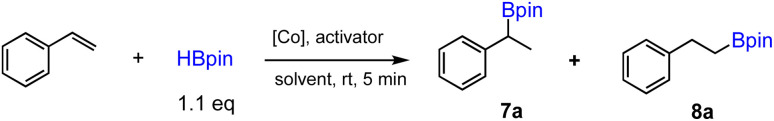					
Entry	Precatalyst	Activator	Solvent	Yield^b^ (%)	Ratio^c^ (*b*/*l*)
1	1	KO^*t*^Bu	THF	98	9 : 1
2	2	KO^*t*^Bu	THF	80	8 : 1
3	3	KO^*t*^Bu	THF	17	10 : 1
4	4	KO^*t*^Bu	THF	35	8 : 1
5	5	KO^*t*^Bu	THF	41	9 : 1
6	6	KO^*t*^Bu	THF	<2	—
7	1	KO^*t*^Bu	Neat	50	3 : 1
8	1	KO^*t*^Bu	Toluene	<1	—
9	1	KO^*t*^Bu	CH_2_Cl_2_	2	—
10	1	KO^*t*^Bu	Et_2_O	62	9 : 1
11	1	KO^*t*^Bu	DMSO	58	5 : 1
12	1	NaO^*t*^Bu	THF	83	6 : 1
13	1	LiO^*t*^Bu	THF	20	3 : 1
14	1	KOCH_3_	THF	72	5 : 1
15	1	K_2_CO_3_	THF	59	8 : 1
16	1	LiNTf_2_	THF	2	—
17	1	NaHBEt_3_	THF	88	5 : 1
18	—	KO^*t*^Bu	THF	0	—
19	1	—	THF	2	—
20	Co(BF_4_)_2_	KO^*t*^Bu	THF	0	—
21^d^	1	KO^*t*^Bu	THF	<5	—
22^e^	1	KO^*t*^Bu	THF	97	9 : 1

a Conditions: styrene (1.0 mmol), HBpin (1.1 mmol), precatalyst (0.025 mol%), activator (1 mol%) and solvent (0.5 mL), 25 ^°^C, 5 min, N_2_.

b Yield of 7a + 8a, determined by GC analysis with hexamethylbenzene as an internal standard.

c Ratio (*b*/*l* = 7a :  8a) determined by GC analysis.

d Reaction run in the air.

e Reaction run using 0.025 mol% of microcrystalline sample of 1.

Having established the ability of 1 as the best-performing precatalyst among six homoleptic complexes, we further screened the influence of reaction conditions such as solvents and activators on the catalytic performance. The solvent effect proved to be significant (entries 7–11). Much lower yield and regioselectivity were observed when the reaction was conducted without a solvent. Toluene and dichloromethane were incompatible solvents for this reaction, as only trace amount of product has been detected for reactions in these solvents. Diethyl ether and dimethylsulfoxide are relatively better, yet still inferior to THF in terms of yield and selectivity. Next, we examined the reaction with different activators (entries 12–17). The results suggest the key role played by KO^*t*^Bu as an activator for both high yield and regioselectivity, while other common activators such as NaO^*t*^Bu, K_2_CO_3_ and NaHBEt_3_ all led to relatively poor results. Finally, control experiments were carried out to show that both cobalt complex and an activator are required to initiate the reaction (entries 18 and 19). It is also worth noting that when Co(BF_4_)_2_ was used instead complex 1 as a precatalyst, no hydroboration of styrene was detected (entry 20). Under the optimal conditions with 1/KOtBu as catalyst, the reaction did not proceed when it was exposed to the air, indicating that a highly air-sensitive reactive intermediate (likely a cobalt hydride species) must have formed and was responsible for the high-efficiency catalysis (entry 21). It is worth mentioning that to achieve the high TOF and regioselectivity, a single crystalline sample was not necessary. Instead, microcrystalline material of 1 could be synthesized in a gram-scale within 1–2 h by simply mixing concentrated, equimolar solutions of pytpy and Co(BF_4_)_2_·6H_2_O in CH_2_Cl_2_ and MeOH, respectively (see the ESI†), and the hydroboration of styrene using this microcrystalline sample was found to be equally efficient (entry 22).

**Scheme 3:**
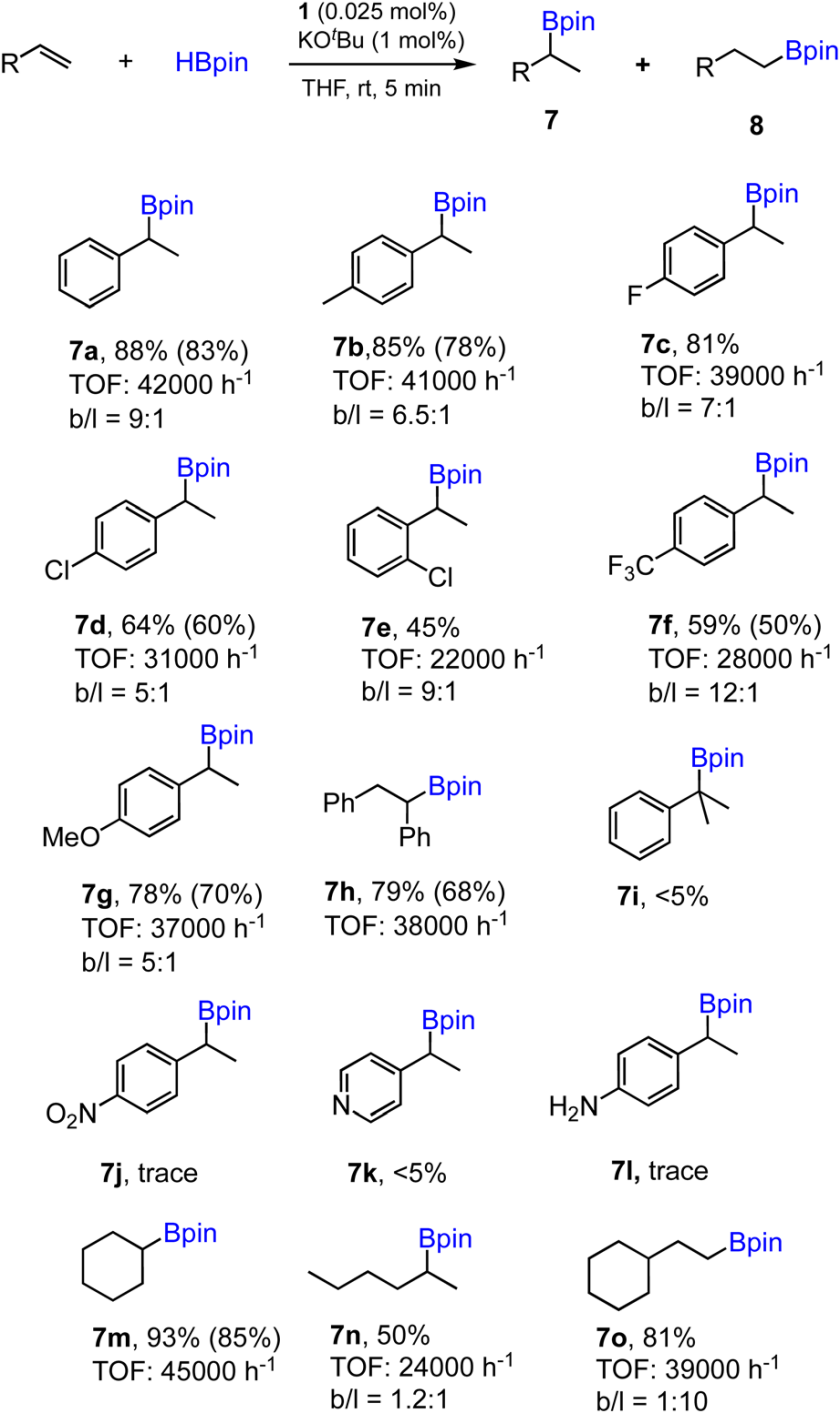
Substrate scope for the hydroboration of alkenes using 1/KO^*t*^Bu. Conditions: alkene (1.0 mmol), HBpin (1.1 mmol), 1 (0.025 mol%), KO^*t*^Bu (1 mol%) and THF (0.5 mL), 25 °C, 5 min, N_2_. Yields of products 7 and the ratio 7/8 (*b*/*l*) determined by GC analysis with hexamethylbenzene as an internal standard. TOF determined based on the yield of regioisomer 7 only. Yields of isolated products given in parentheses.

To further demonstrate the effectiveness of 1 as a precatalyst for other alkene substrates, we employed the optimized conditions (entry 1, Table 1) to examine substituted and functionalized styrenes. The results for a range of substrates tested are summarized in Scheme 3. First, methyl- and fluoro-substituted styrenes are suitable substrates affording the corresponding alkylboronates with high TOFs and slightly lower regioselectivity. Product 7b was readily isolated from the mixture with 80% yield. However, when 4-chloro- or 2-chlorostyrene was used, the reaction ran slower and moderate yields were obtained in 5 min for both cases. Lower TOF (28 000 h^−1^) was also found in the case of 4- trifluoromethylstyrene as a substrate. Styrene with an electron-donating 4-methoxy group proceeded well with good yield, while the regioselectivity dropped to 5 : 1 (7g). cis-Stilbene was found to be active substrate for hydroboration to afford 7h with appreciable isolated yield and high TOF. However, 1,1-disubstituted alkene shows poor reactivity under standard conditions (7i). Styrenes containing functional groups such as nitro, amino or pyridyl are inactive substrates, similar to the results reported previously using polymeric [Co(pytpy)Cl_2_]_*n*_ as a precatalyst.^8*a*^ In addition, aliphatic and cyclic alkenes are also reactive substrates, however, poor regioselectivity was obtained for 1-hexene (7n) and anti-Markovnikov selectivity was found for vinylcyclohexane (7o).

Next, we explored the functional group tolerance of 1/KO^*t*^Bu system for styrene hydroboration by adding a second reducible substrate, which is so called a fast catalyst robustness screening.^22^ Thus, styrene was chosen to react with HBpin under standard conditions in the presence of equimolar additives as listed in Table 2. Both ketone and aldehyde showed strongly competing reactions with styrene, as 60% ketone and 95% aldehyde hydroboration was detected as the major reactions, respectively (entries 1 and 2, Table 2). The ester showed little influence on the efficiency of styrene hydroboration with no change on the regioselectivity (entry 3). However, both amide and nitrobenzene have completely suppressed the reactions (entries 4 and 5). Interestingly, butyronitrile is compatible with the reaction, while increasing the regioselectivity to 15 : 1 (entry 6). Finally, the presence of styrene oxide significantly decreased the yield of 7 albeit the regioselectivity remained. These results indicate somewhat inferior functional group tolerance of homoleptic complex 1, compared to the polymeric precatalyst [Co(pytpy)Cl_2_]_*n*_.^8*a*,14^

**Table 2:** Catalyst robust screening experiments^a^

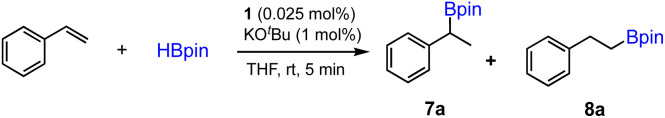			
Entry	Additive	Yield (7a + 8a)^b^/%	Ratio (7a/8a)^b^
1^c^	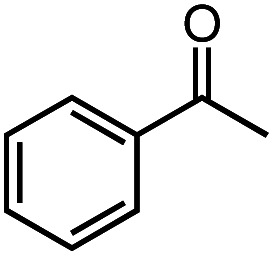	40%	8 : 1
2^d^	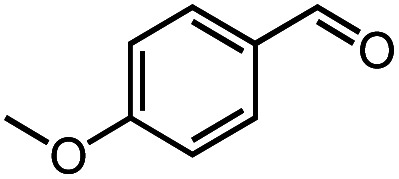	4%	—
3	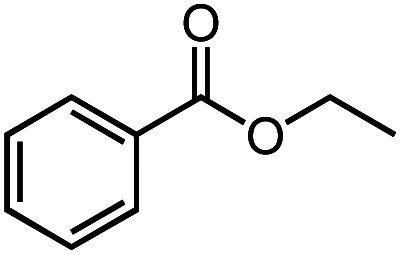	72%	9 : 1
4	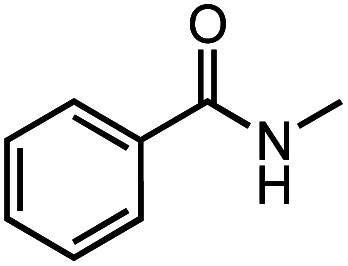	6%	—
5	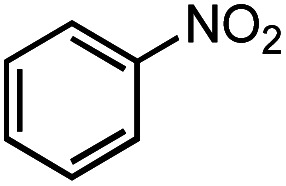	3%	—
6	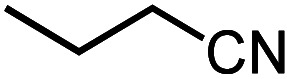	90%	15 : 1
7	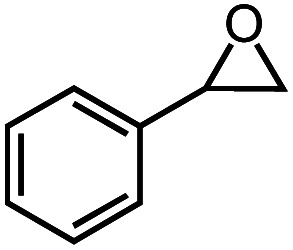	35%	9 : 1

a Conditions: styrene (0.5 mmol), HBpin (0.5 mmol), 1 (0.025 mol%), KO^*t*^Bu (1 mol%), other reducible substrate (0.5 mmol) and THF (1 mL), 25 °C, 5 min, N_2_.

b Determined by GC analysis using hexamethylbenzene as an internal standard.

c 60% ketone hydroboration was detected.

d 95% aldehyde hydroboration was detected.

**Scheme 4:**
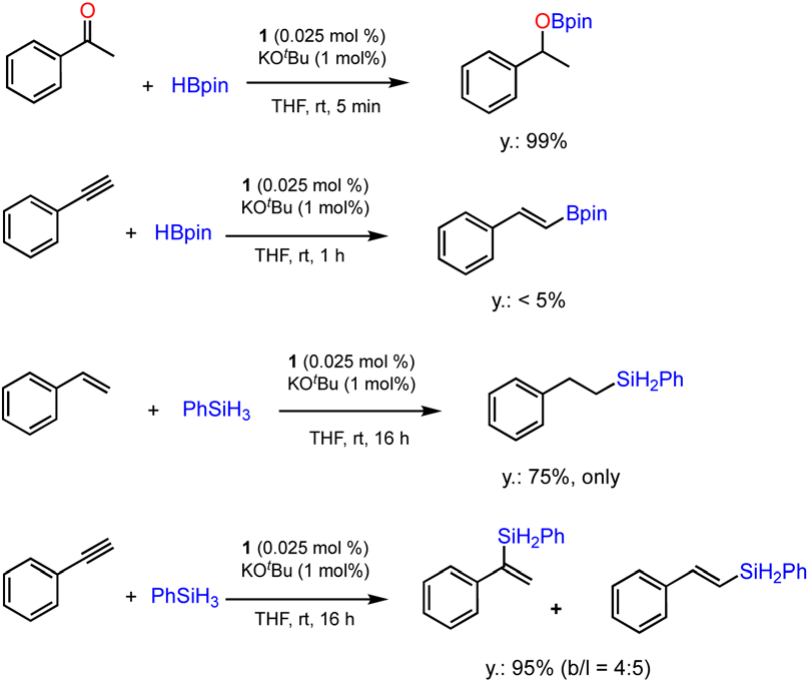
Additional catalytic tests for hydroboration and hydrosilylation reactions using 1. Conditions: substrate (0.5 mmol), HBpin or PhSiH_3_ (0.55 mmol), 1 (0.025 mol%), KO^*t*^Bu (1 mol%) and THF (0.5 mL), 25 °C, N_2_, indicated time. Yields and regioselectivity determined by GC analysis using hexamethylbenzene as an internal standard.

The ability of precatalyst 1 to promote hydroboration and hydrosilylation for several other substrates was further evaluated. The preliminary results are presented in Scheme 4. The hydroboration of ketone was furnished under the standard conditions within 5 min, while phenylacetylene is almost inactive for hydroboration after 1 h. This is in sharp contrast with the results obtained using [Co(pytpy)Cl_2_]_*n*_ as precatalyst where very high TOFs could be achieved.^14*b*^ In addition, hydrosilylation of styrene and phenylacetylene using phenylsilane as a Si–H source has been investigated. It was found that styrene has experienced effective hydrosilylation to afford the anti-Markovnikov product with complete regioselective control. However, hydrosilylation of phenylacetylene was accomplished within 16 h in 95% yield with poor regioselectivity (*b*/*l* = 4 : 5). The results indicate that both reactivity and regioselectivity are highly substrate-dependent when using 1/KO^*t*^Bu as the catalyst.

## Conclusions

In summary, we have synthesized and characterized homoleptic complexes of cobalt or iron with pytpy ligand. The direct reaction of pytpy and metal tetrafluoroborate resulted in the monoprotonation at the non-coordinating pyridyl moiety, namely [M(pytpy)(H-pytpy)][BF_4_]_3_ (M = Co or Fe). X-ray structural analysis reveals the conformational bending of these complexes in the solid state due to the formation of 1-D hydrogen-bonded chains, in comparison with the common complex, Co(pytpy)_2_(BF_4_)_2_. These new complexes (1–3) along with several known analogues (4–6) have been explored as precatalysts for the regioselective hydroboration of alkenes. The best catalytic system 1/KO^*t*^Bu was found to furnish the hydroboration of styrene with a very high TOF of ∼47 000 h^−1^, comparable to the most efficient precatalyst [Co(pytpy)Cl_2_]_*n*_ reported thus far. The method can be applied to a range of styrene derivatives for the regioselective synthesis of branched alkylboronates. However, some limitation of substrates has been disclosed. Catalyst robustness screening experiments provide further insights into the functional group tolerance of current catalyst. Preliminary experiments on relevant substrates for hydroboration and hydrosilylation catalysis were also conducted to extend its applicability for other conversions.

## Experimental

### General

Unless specified otherwise, all reactions were carried out under a dry N_2_ atmosphere using standard glovebox and Schlenk techniques. Anhydrous grade solvents and reagents used were obtained from Aldrich or Fisher Scientific and stored over 4 Å molecular sieves. All chemicals of analytical grade including the alkene substrates and additives are used as received from Alfa Aesar, Acros, TCI America or Fisher Scientific without further purification. FT-IR spectra were recorded on a Shimadzu 8400S instrument with solid samples under N_2_ using a Golden Gate ATR accessory. Elemental analyses were performed by Midwest Microlab LLC in Indianapolis in the US. ^1^H NMR and ^13^C NMR spectra were obtained at room temperature on a Bruker AV 400, 500 or 600 MHz NMR spectrometer, with chemical shifts (*δ*) referenced to the residual solvent signal. HR-MS data were obtained on an Agilent 6550 QToF coupled to an Agilent 1290 Infinity LC system. GC-MS analysis was obtained using a Shimadzu GCMS-QP2010S gas chromatograph mass spectrometer (column: SHRX1-5MS, thickness: 0.25 m, diameter: 0.25 mm, length: 30.0 m; conditions: 30–200 °C, 10 °C min^−1^, injection temperature: 100 °C; solvent cutoff: 3 min).

### Synthesis of 1

A solution of pytpy (31.0 mg, 0.100 mmol) in MeOH/CH_2_Cl_2_ (10 mL, 1 : 3, v/v) was placed in a test tube. A blank solution of MeOH/CH_2_Cl_2_ (4 mL, 1 : 1, v/v) was layered on the top of the ligand solution, followed by a solution of Co(BF_4_)_2_·6H_2_O (34.1 mg, 0.100 mmol) in MeOH (8 mL). The tube was sealed and allowed to stand at room temperature for about four weeks, during which time X-ray quality yellow blocks grew at the bottom of the tube. The crystals were collected by decanting the solvent and washed with MeOH and then dried *in vacuo*. Yield: 43.2 mg (92% based on pytpy). FT-IR (solid, cm^−1^): 3078m, 1620m, 1597s, 1538s, 1470s, 1428s, 1405s, 1247s, 1031br, 822s, 786s, 733m. ^1^H NMR (500 MHz, DMSO) *δ* 83.31 (bs), 48.22 (bs), 31.38 (bs), 30.84 (bs), 12.82 (bs), 10.49 (bs), 9.87 (bs) ppm. HR-MS (ESI positive): 679.1766 ([M-3(BF_4_^−^)-H^+^], Cald. 679.1769). Anal. Calcd. for C_40_H_29_B_3_CoF_12_N_8_: C 51.05, H 3.11, N 11.91%. Found C 50.82, H 3.01, N 11.69. In a separate experiment, in a 100 mL flask, pytpy (0.62 g, 2.00 mmol) was dissolved in MeOH/CH_2_Cl_2_ (40 mL, 1 : 3, v/v), to which a solution of Co(BF_4_)_2_·6H_2_O (0.68 g, 2.00 mmol) in MeOH (20 mL) was added dropwise in 3 min. The reaction mixture was allowed to stay for an additional 1.5 h, red microcrystals had formed and were filtered to give bulk sample of 1 (yield: 0.83 g, 88%). Catalytic experiment confirmed its activity and efficiency for styrene hydroboration (entry 22, Table 1).

### Synthesis of 2

pytpy (31.0 mg, 0.100 mmol) was dissolved in MeOH/CH_2_Cl_2_ (8 mL, 1 : 3, v/v) in a 20 mL vial, to which was added a solution of CoCl_2_·6H_2_O (11.9 mg, 0.050 mmol) in MeOH (3 mL). The mixture was stirred for 15 min at ambient temperature and then a solution of NaBF_4_ (66.0 mg, 0.600 mmol) in MeOH (2 mL) was added, the resulting precipitate was filtered, washed with MeOH and dried *in vacuo*. X-ray quality crystals were obtained by slow diffusion of diethyl ether into a concentrated solution of 2 in acetonitrile. Yield: 34.5 mg (81%). FT-IR (solid, cm^−1^): 3057m, 1619m, 1597s, 1538s, 1470s, 1407s, 1245s, 1053br, 896m, 823m, 789s, 732m. ^1^H NMR (400 MHz, DMSO) *δ* 83.42 (bs), 48.26 (bs), 31.69 (bs), 30.80 (bs), 12.82 (bs), 10.43 (bs), 9.92 (bs) ppm. HR-MS (ESI positive): 679.1766 ([M-2(BF_4_^−^)], Cald. 679.1769). Anal. Calcd. for C_40_H_28_B_2_CoF_8_N_8_: C 56.31, H 3.31, N 13.13%. Found C 56.05, H 3.19, N 13.04.

### Synthesis of 3

The procedure is similar to that for 1, except that Fe(BF_4_)_2_·6H_2_O (33.8 mg, 0.100 mmol) was used. Brown plate-like crystals of 3 were collected in 85% yield (40 mg). FT-IR (solid, cm^−1^): 3603w, 3540w, 3075m, 1595s, 1537s, 1482m, 1467m, 1408s, 1286m, 1246m, 1053br, 895m, 821s, 788s, 755s, 733m. HR-MS (ESI positive): 676.1774 ([M-3(BF_4_^−^)-H^+^], Cald. 676.1780).

### General procedure for 1-catalysed alkene hydroboration

In a glovebox under N_2_ atmosphere, cobalt catalyst 1 (0.23 mg, 0.25 μmol, 0.025 mol%) and KO^*t*^Bu (1.12 mg, 1 mol%) was dissolved in THF (0.5 mL) in a 3.8 mL glass vial equipped with a small stir bar. The mixture was stirred for 1 min. Alkenes (1.0 mmol) and pinacolborane (141 mg, 1.1 mmol) were then added. The reaction mixture was allowed to stir at room temperature for 5 min and then the reaction was quenched by exposing the reaction solution to air and adding CH_2_Cl_2_ (1 mL) to the solution. The crude reaction mixture was first analyzed by GC-MS to determine the total yields of desired alkylboronates and the ratio of the regioisomeric products by comparing the GC traces with those of authentic samples.^8*a*^ The reaction mixture for several selected products was then evaporated under reduced pressure and the product was purified through a SiO_2_ column chromatography using ethyl acetate/hexane as an eluent. The pure alkylboronates of major products were characterized by ^1^H and ^13^C NMR spectroscopies.

### Catalyst robustness screening

In a glovebox under N_2_ atmosphere, cobalt precatalyst 1 (0.12 mg, 0.025 mol%) and KO^*t*^Bu (1.12 mg, 1 mol%) was dissolved in THF (0.5 mL) in a 3.8 mL glass vial equipped with a stir bar. The mixture was stirred for 1 min. Styrene (52 mg, 0.5 mmol), additive (0.5 mmol) and pinacolborane (64 mg, 0.5 mmol) were then added sequentially. Hexamethylbenezene (25 mg) was added as an internal standard for GC analysis. The reaction mixture was allowed to stir at room temperature for 5 min. The reaction was quenched by exposing the reaction solution to air and adding CH_2_Cl_2_ (1 mL) to the solution. The crude product was analyzed by GC-MS to determine the GC yield and ratio of the regioisomeric products from styrene hydroboration. In each case, the identification of the corresponding boronate esters have been made by comparing their GC retention time and MS data with the authentic samples.^8*a*^

### X-ray crystallography

X-ray diffraction data were collected on a Bruker X8 Kappa Apex II diffractometer using Mo Kα radiation (for 1) or on a Bruker D8 VENTURE diffractometer using Cu Kα radiation (for 2 and 3). Crystal data, data collection and refinement parameters are summarized in Table S1 (ESI†). The structures were solved using a dual-space method and standard difference map techniques and were refined by full-matrix least-squares procedures on *F*^2^ with SHELXTL.^23^ All hydrogen atoms bound to carbon were placed in calculated positions and refined with a riding model [*U*_iso_(H) = 1.2 − 1.5*U*_eq_(C)]. The hydrogen atom bound to nitrogen was located on the difference map and refined freely or with an N–H distance restraint (for 3) [*U*_iso_(H) = 1.2*U*_eq_(N)].

## Author contributions

G. Z. and S. Z. conceived and supervised the project. H. Z., N. Z. and C. M. performed experimental studies. M. C. N. conducted the X-ray crystallographic analysis. All authors analysed the data and wrote the article.

## Conflicts of interest

There are no conflicts to declare.

## Supplementary Material

RA-013-D3RA06113B-s001

RA-013-D3RA06113B-s002
