# Supplementary material for: Homoleptic octahedral CoII complexes as precatalysts for regioselective hydroboration of alkenes with high turnover frequencies
Source: RSC Adv. 2023 Sep 22;13(40):28089–96. doi: 10.1039/d3ra06113b (PMC10517108; doi:10.1039/d3ra06113b)
Supplement: RA-013-D3RA06113B-s001 [file RA-013-D3RA06113B-s001.pdf]

Electronic Supplementary Material (ESI) for RSC Advances.
This journal is © The Royal Society of Chemistry 2023

Electronic Supplementary Information:

Homoleptic octahedral Co^{II} complexes as precatalysts for regioselective hydroboration of alkenes with high turnover frequencies

Guoqi Zhang,*[,a] Haisu Zeng,[a,b] Nora Zadori,[a] Camila Marino,[a] Shengping Zheng,[b] and Michelle C. Neary[b]

[a] Department of Sciences, John Jay College and Ph.D. Program in Chemistry, The Graduate Center of the City University of New York, New York, 10019 NY, USA.

[b] Department of Chemistry, Hunter College, the City University of New York, New York, 10065 NY, USA.

Table S1. X-ray crystallographic data for **1-3**.

	**1**•CH_2Cl_2	**2**•CH_3CN	**3**
lattice	Monoclinic	Monoclinic	Monoclinic
formula	$C_{41}H_{31}B_3Cl_2CoF_{12}N_8$	$C_{42}H_{31}B_2CoF_8N_9$	$C_{40}H_{29}B_3F_{12}FeN_8$
formula weight	1026.00	894.31	937.99
space group	$P2_1/c$	Pc	$P2_1/c$
a/Å	14.147(4)	8.7498(3)	14.1345(6)
b/Å	16.117(4)	25.9051(8)	15.9916(7)
c/Å	18.997(5)	8.5927(3)	18.7291(8)
α/°	90	90	90
β/°	105.971(5)	90.761(2)	105.142(3)
γ/°	90	90	90
V/Å^3	4164.2(18)	1947.49(11)	4086.4(3)
Z	4	2	4
temperature (K)	130(2)	130(2)	130(2)
radiation (λ, Å)	0.71073	1.54178	1.54178
ρ (calcd.) g cm^{-3}	1.637	1.525	1.525
μ (Mo/Cu Kα), mm^{-1}	0.639	4.192	3.827
θ max, deg.	31.240	74.507	72.104
no. of data collected	129751	48092	79057
no. of data	13527	7602	7970
no. of parameters	639	601	615
R_1 [$I > 2\sigma(I)$]	0.0569	0.0553	0.1048
wR_2 [$I > 2\sigma(I)$]	0.1465	0.1335	0.2103
R_1 [all data]	0.0787	0.0613	0.1342
wR_2 [all data]	0.1596	0.1373	0.2263
GOF	1.091	1.073	1.082
R_{int}	0.0512	0.1115	0.1852

Characterization data of isolated products.

7a[1]: Colorless oil. Yield: 192 mg (83%). ^{1}H NMR (600 MHz, $CDCl_3$) δ 7.33 – 7.25 (m, 4H), 7.17 (tt, J = 6.8, 1.5 Hz, 1H), 2.48 (q, J = 7.5 Hz, 1H), 1.38 (d, J = 7.5 Hz, 3H), 1.24 (d, J = 8.1 Hz, 12H) ppm; ^{13}C NMR (151 MHz, $CDCl_3$) δ 208.4, 145.0, 128.4, 127.9, 125.1, 83.3, 24.4, 17.0 ppm. GC-MS (m/z): 232 (calc. 232).

7b[1]: Colorless oil. Yield: 191 mg (78%). ^{1}H NMR (600 MHz, $CDCl_3$) δ 7.13 (d, J = 8.1 Hz, 2H), 7.10 – 7.02 (m, 2H), 2.41 (q, J = 7.6 Hz, 1H), 2.32 (d, J = 2.3 Hz, 3H), 1.33 (d, J = 7.6 Hz, 3H), 1.26 – 1.16 (m, 12H) ppm; ^{13}C NMR (151 MHz, $CDCl_3$) δ 141.9, 134.4, 129.0, 127.7, 83.2, 29.5, 24.6, 21.0, 17.3 ppm. GC-MS (m/z): 246 (calc. 246). t_r = 14.9 min (major), t_r = 16.1 min (minor). Note: signals for a small amount of the minor regioisomer were still observed in the NMR spectra after purification by column chromatography.

7d[1]: Colorless oil. Yield: 160 mg (60%). ^{1}H NMR (500 MHz, $CDCl_3$) δ 7.22 (d, J = 8.5 Hz, 2H), 7.15 (d, J = 8.5 Hz, 2H), 2.41 (q, J = 7.5 Hz, 1H), 1.32 (d, J = 7.5 Hz, 3H), 1.21 (d, J = 5.6 Hz, 12H) ppm; ^{13}C NMR (126 MHz, $CDCl_3$) δ 143.5, 130.7, 129.1, 128.4, 83.4, 29.4, 24.6, 24.6, 17.0 ppm. GC-MS (m/z): 266 (calc. 266).

7f[1]: While solid of **2i**. Yield: 150 mg (50%). ^{1}H NMR (500 MHz, $CDCl_3$) δ 7.41 (s, 2H), 7.24 (d, J = 8.2 Hz, 2H), 2.42 (q, J = 7.5 Hz, 1H), 1.27 (d, J = 7.5 Hz, 3H), 1.13 (s, 12H) ppm; ^{13}C NMR (126 MHz, $CDCl_3$) δ 149.3, 134.0, 128.0, 127.4 (q, J = 32.2 Hz), 125.2 (q, J = 3.8 Hz), 124.5 (q, J = 271.8 Hz), 83.6, 29.9, 24.6, 16.7 ppm. GC-MS (m/z): 300 (calc. 300).

7g[1]: Yellowish oil. Yield: 183 mg (70%). ^{1}H NMR (600 MHz, $CDCl_3$) δ 7.15 (d, J = 8.7 Hz, 2H), 6.82 (d, J = 8.8 Hz, 2H), 3.77 (s, 3H), 2.38 (q, J = 7.5 Hz, 1H), 1.31 (d, J = 7.6 Hz, 3H), 1.25 (s, 12H) ppm; ^{13}C NMR (151 MHz, $CDCl_3$) δ 128.6, 113.8, 83.2, 55.2, 29.1, 24.6, 24.6, 17.4 ppm. GC-MS (m/z): 262 (calc. 262). Note: signals for a small amount of the minor regioisomer were still observed in the NMR spectra after purification by column chromatography.

7h[1]: White solid. Yield: 209 mg (68%). ^{1}H NMR (600 MHz, $CDCl_3$) δ 7.17 – 7.12 (m, 4H), 7.12 – 7.06 (m, 4H), 7.02 (ddt, *J* = 6.5, 4.8, 2.1 Hz, 2H), 3.06 (dd, *J* = 13.5, 9.8 Hz, 1H), 2.87 (dd, *J* = 13.5, 6.9 Hz, 1H), 2.60 (dd, *J* = 9.8, 6.9 Hz, 1H), 0.99 (s, 12H) ppm; ^{13}C NMR (151 MHz, $CDCl_3$) δ 142.7, 141.8, 129.0, 128.9, 128.6, 128.5, 128.4, 128.3, 128.2, 128.0, 125.9, 125.5, 83.4, 39.0, 34.5, 24.7.ppm. GC-MS (m/z): 308 (calc. 308).

7m[1]: Colorless oil. Yield: 179 mg (85%). ^{1}H NMR (500 MHz, $CDCl_3$) δ 1.62 – 1.48 (m, 5H, overlapping), 1.29 – 1.18 (m, 6H), 1.16 (s, 12H) ppm; ^{13}C NMR (126 MHz, $CDCl_3$) δ 82.7, 29.7, 28.0, 27.1, 26.8, 24.8 ppm. GC-MS (m/z): 210 (calc. 210).

<u>Copies of NMR spectra</u>:

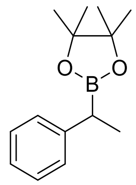

(**7a**)

^{1}H NMR (600 MHz, $CDCl_3$):

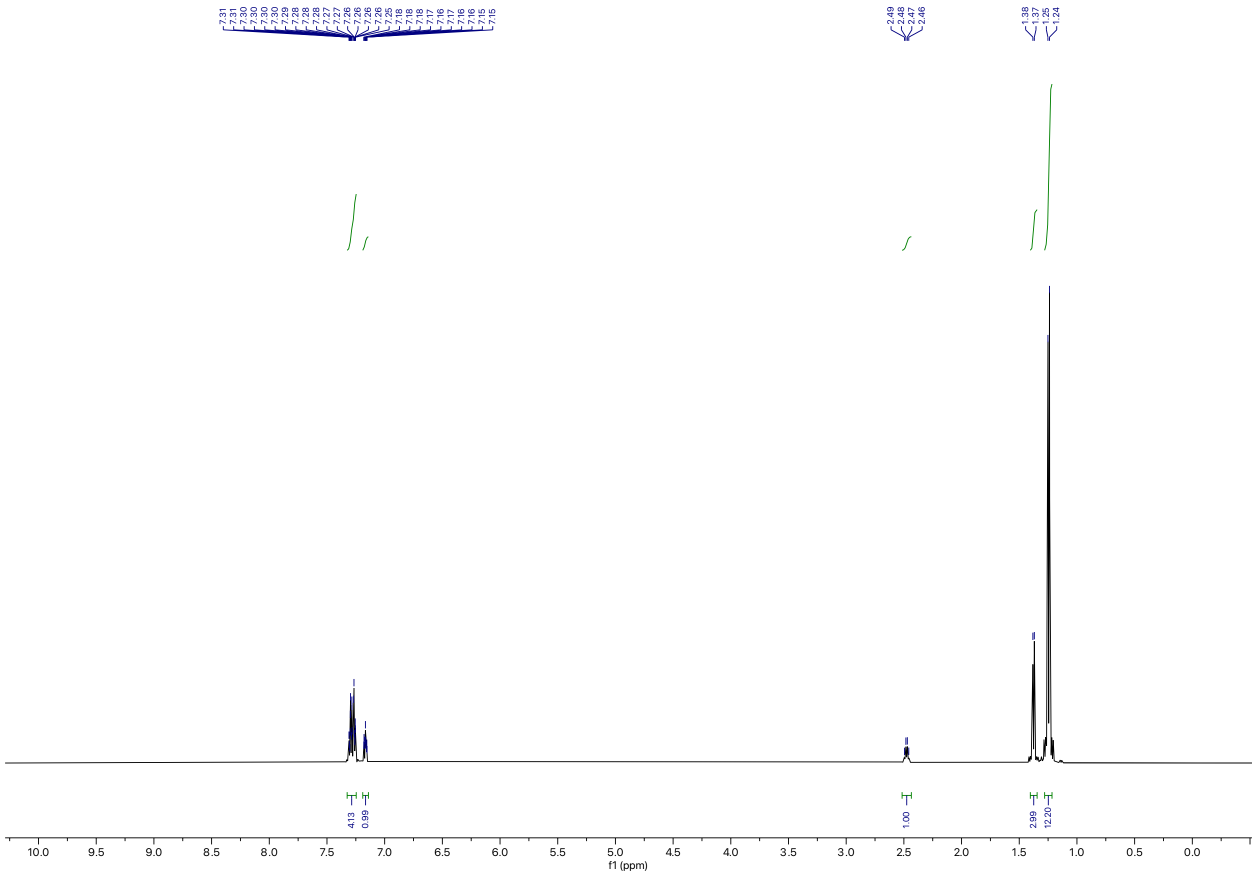

^{13}C NMR (151 MHz, $CDCl_3$):

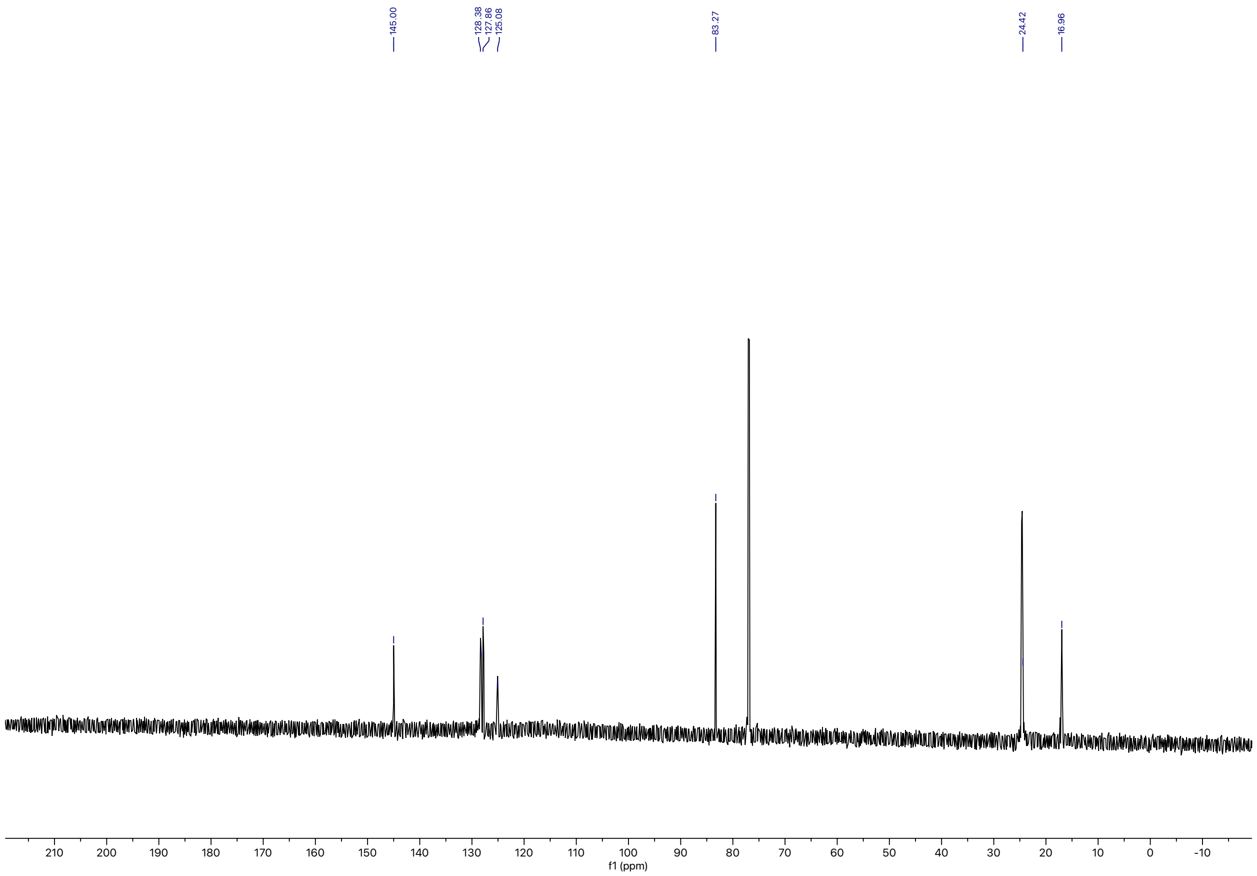

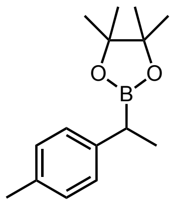

(**7b**)

^{1}H NMR (600 MHz, $CDCl_3$):

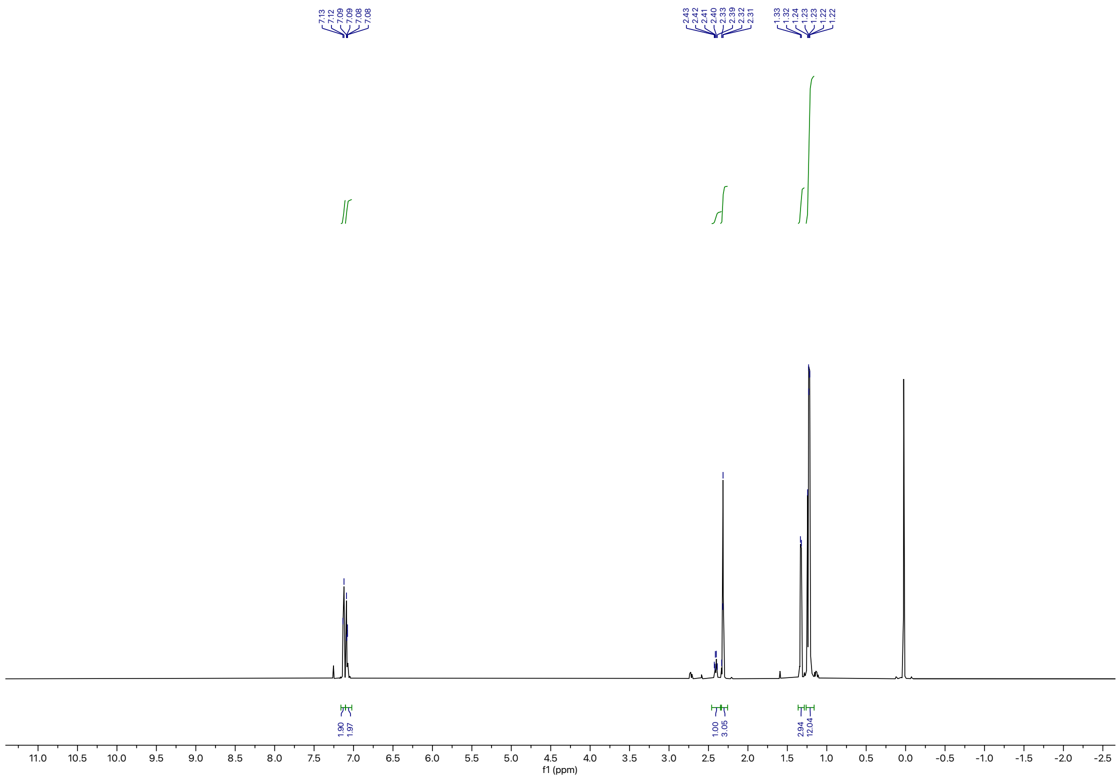

^{13}C NMR (151 MHz, $CDCl_3$):

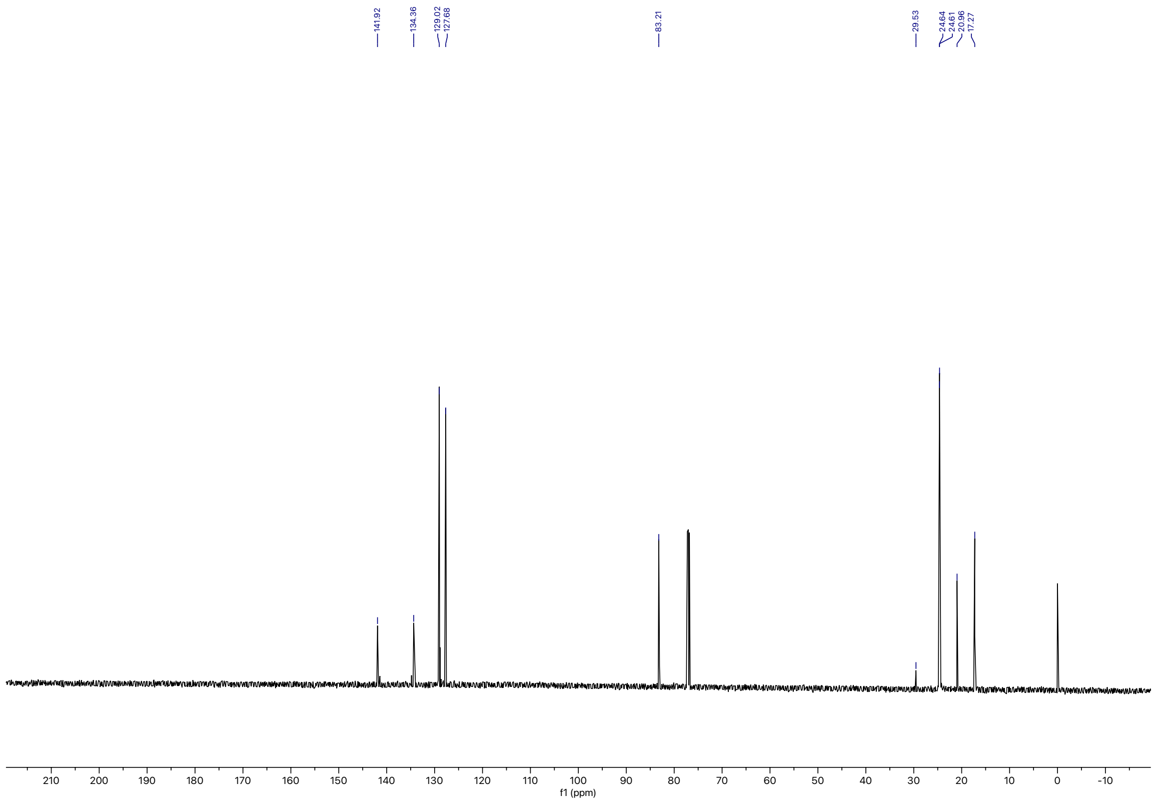

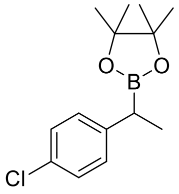

(**7d**)

^{1}H NMR (500 MHz, $CDCl_3$):

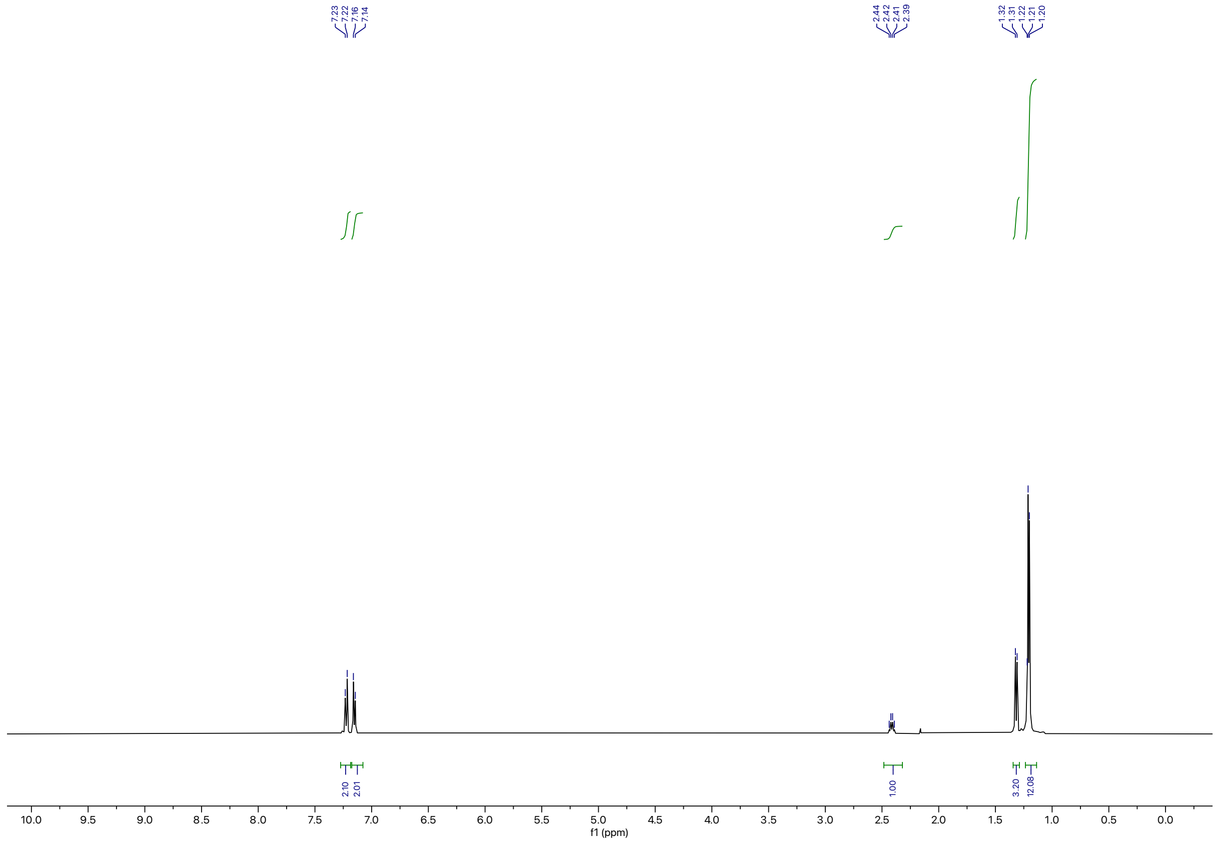

^{13}C NMR (126 MHz, $CDCl_3$):

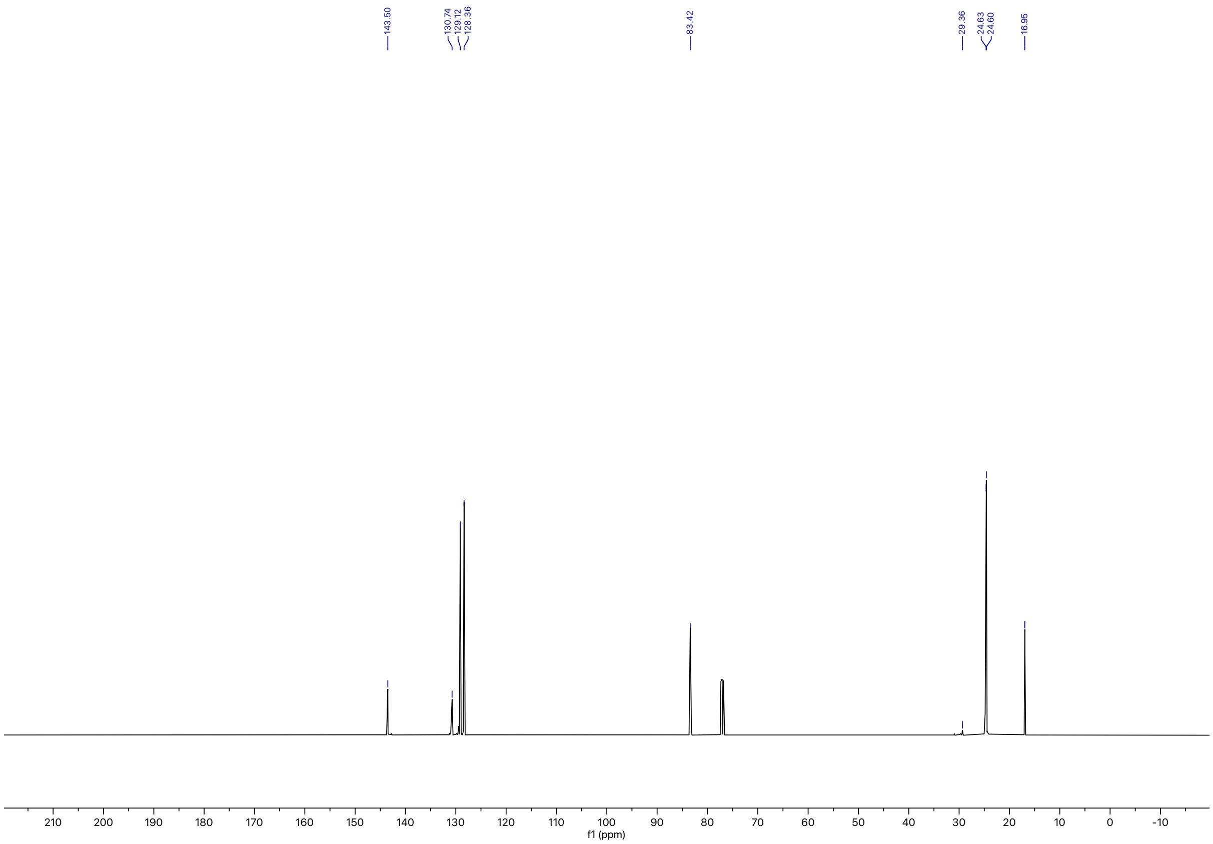

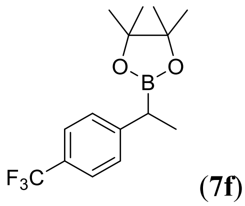

(7f)

^{1}H NMR (500 MHz, $CDCl_3$):

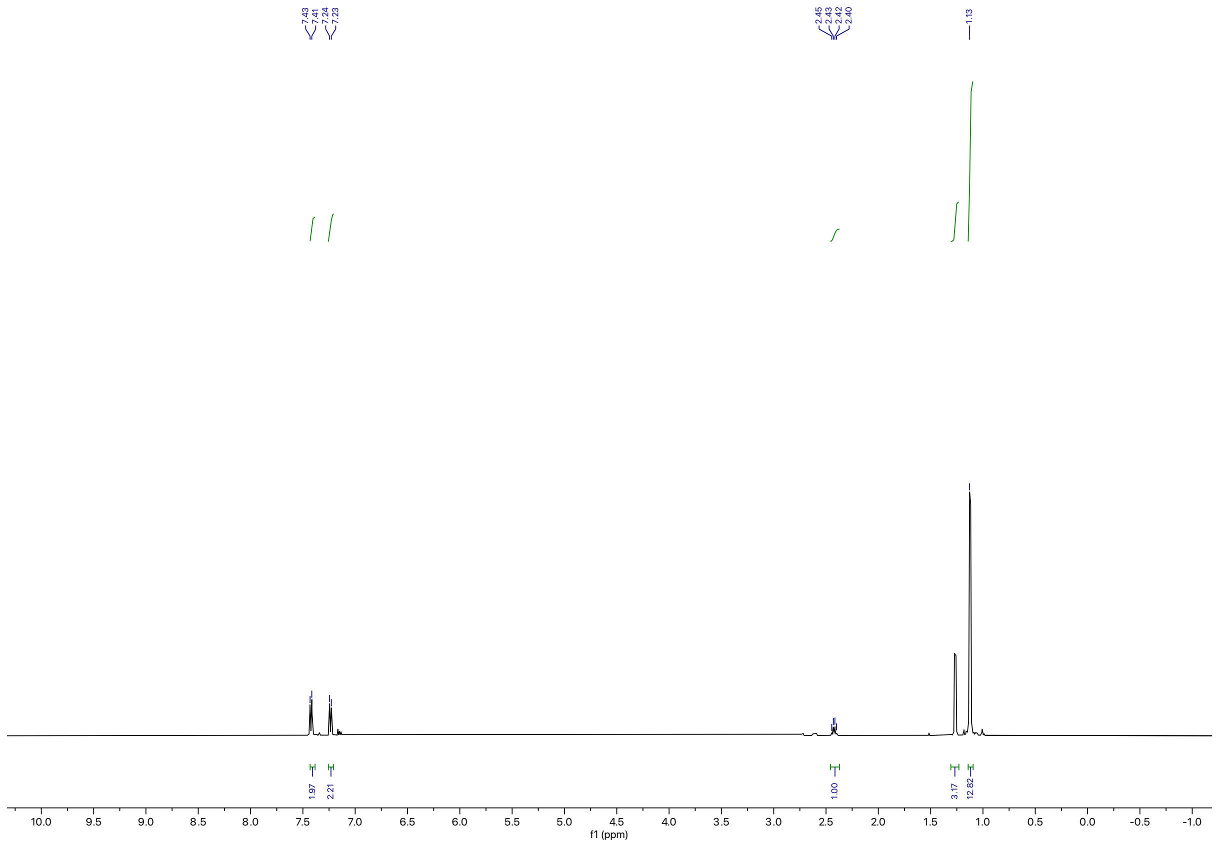

^{13}C NMR (126 MHz, $CDCl_3$):

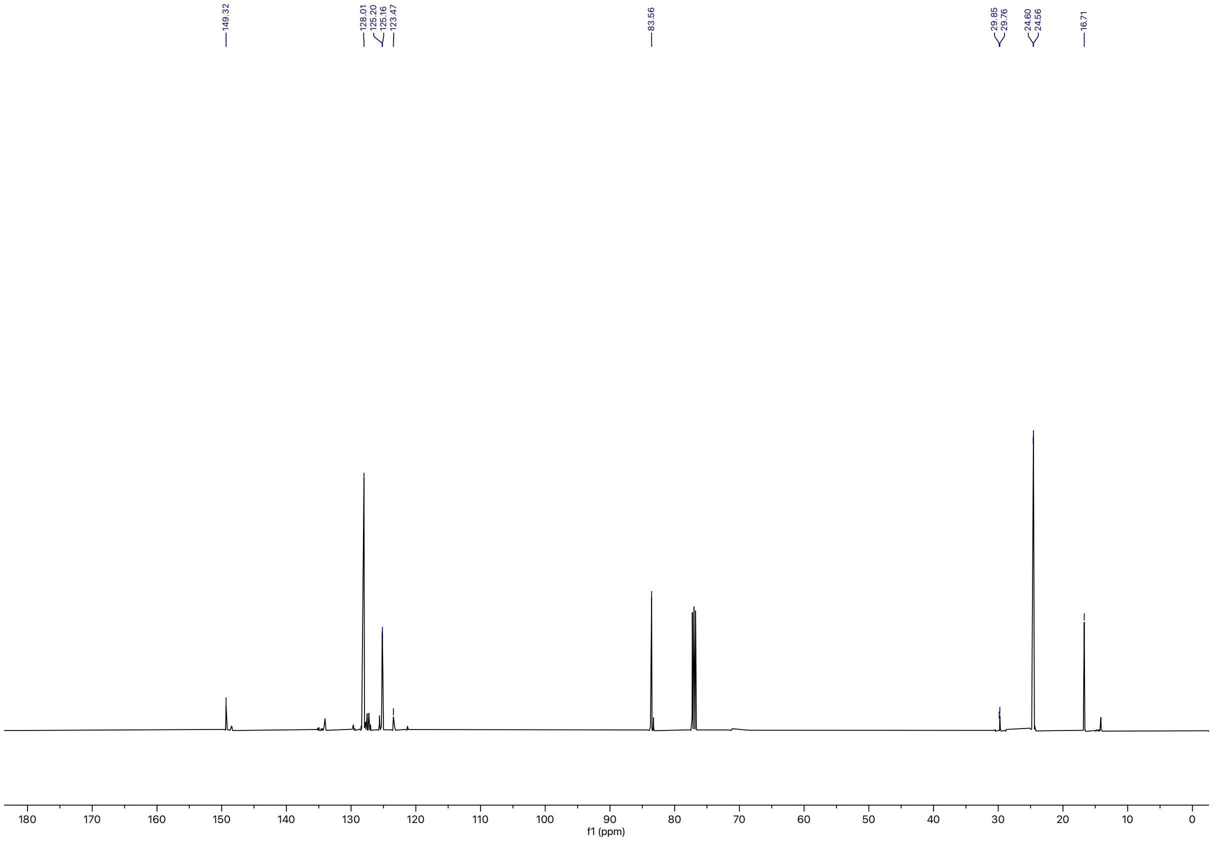

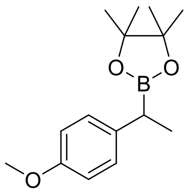

(7g)

^{1}H NMR (600 MHz, $CDCl_3$):

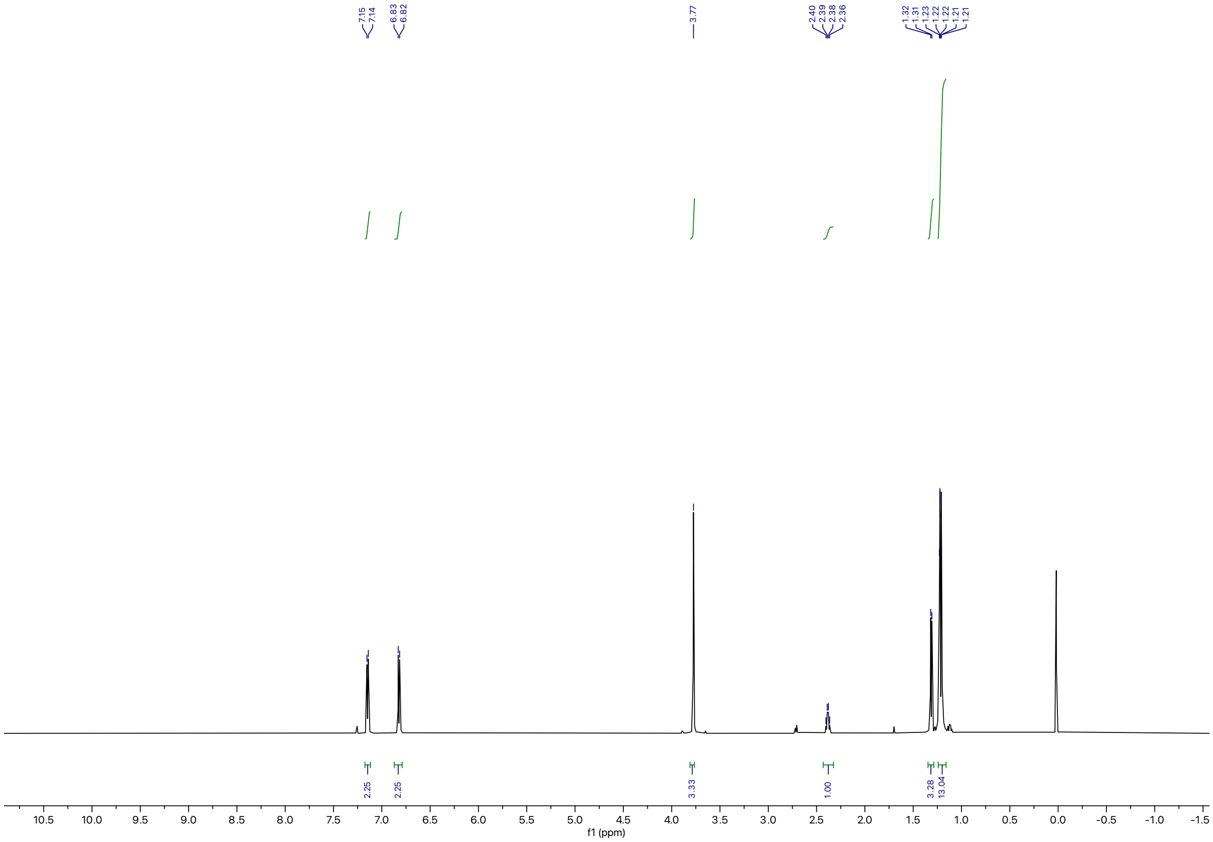

^{13}C NMR (151 MHz, $CDCl_3$):

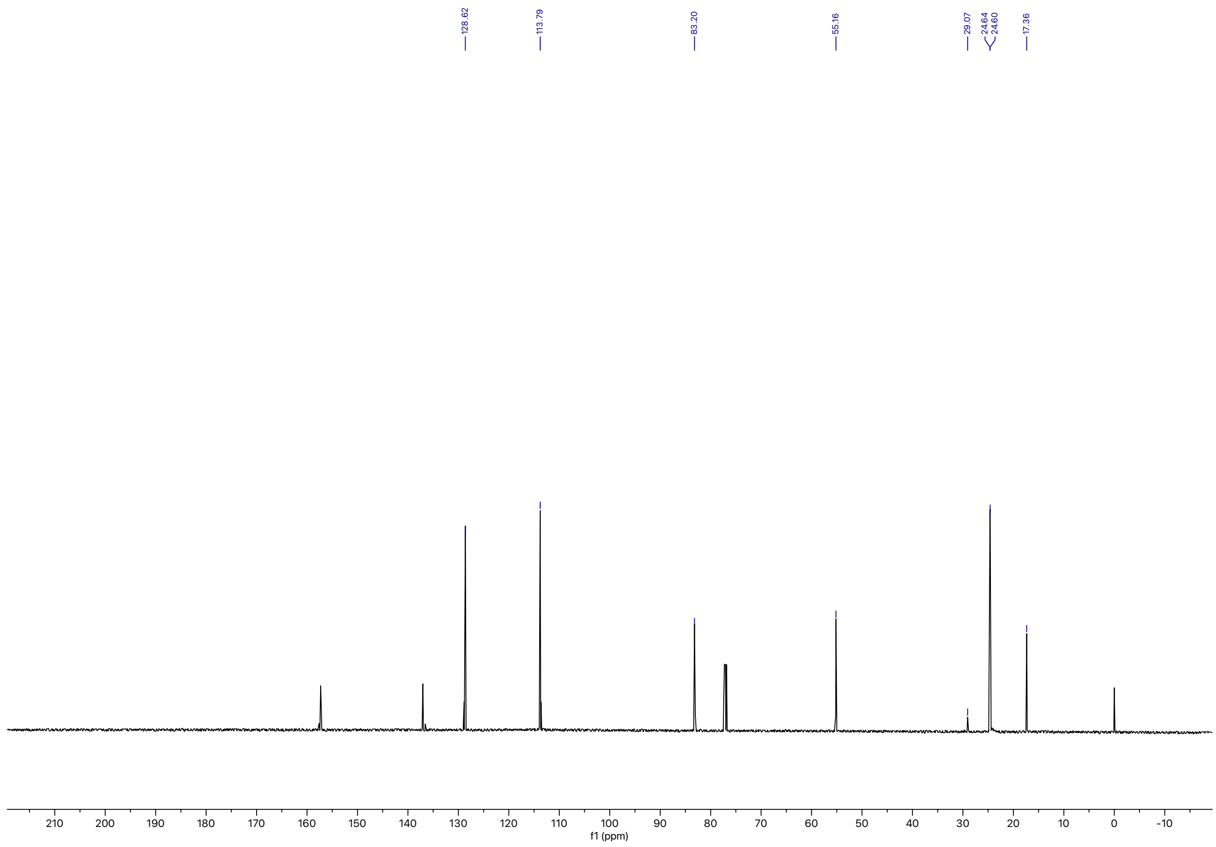

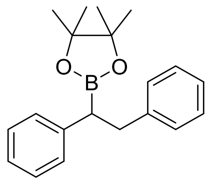

(**7h**)

^{1}H NMR (600 MHz, $CDCl_3$):

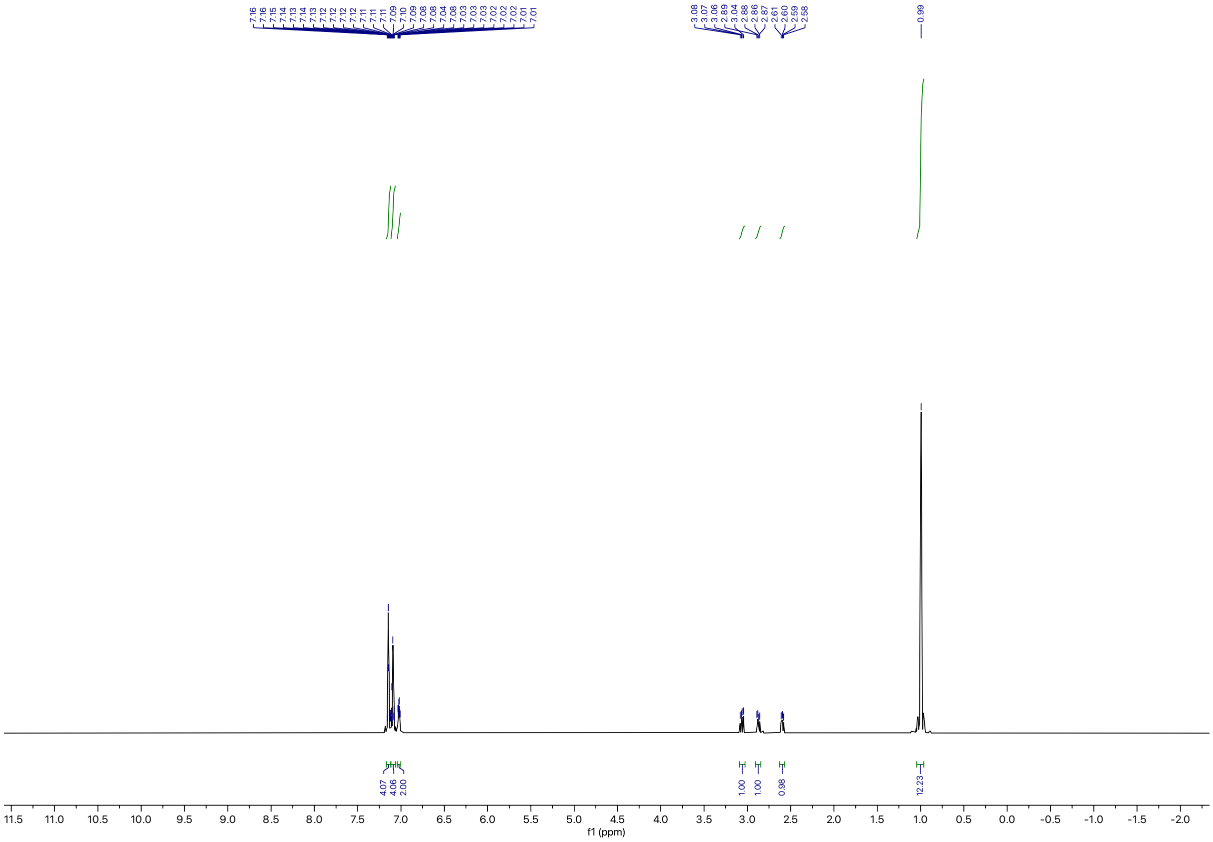

^{13}C NMR (151 MHz, $CDCl_3$):

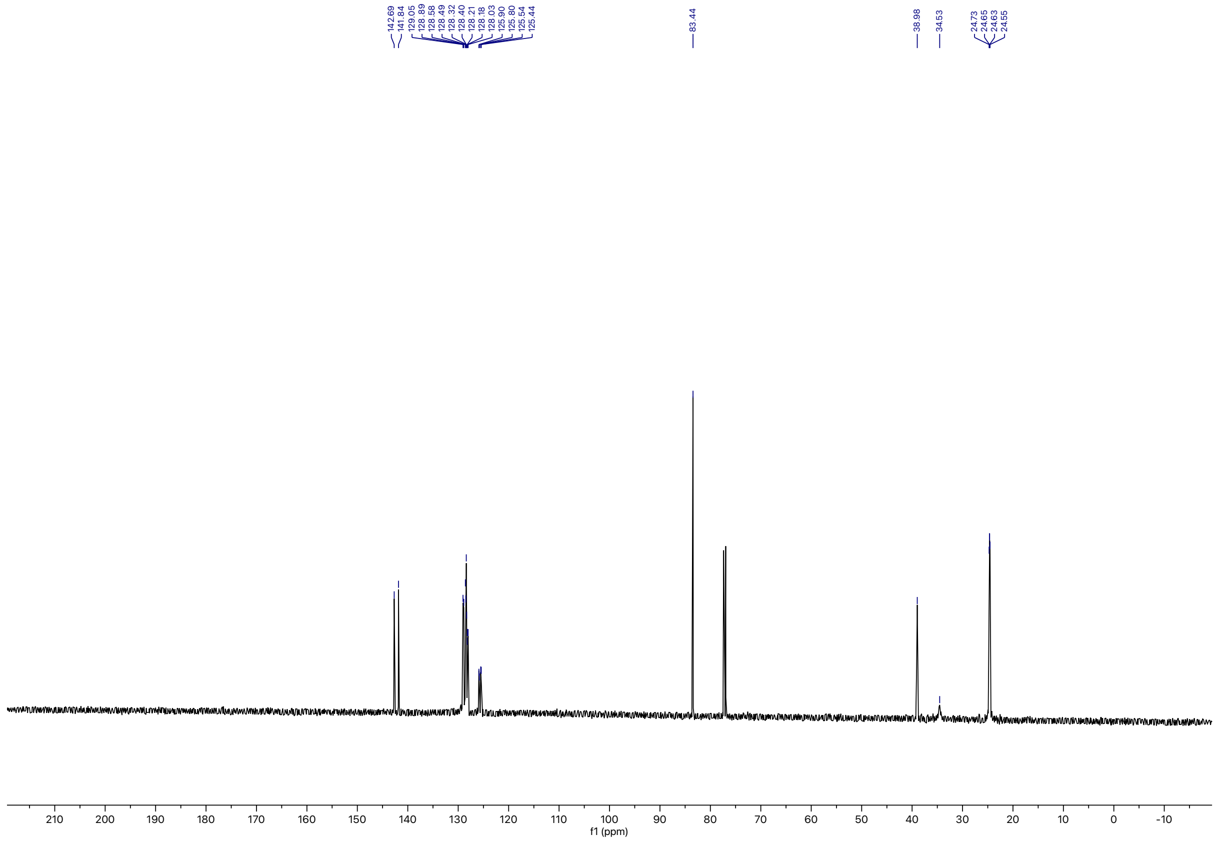

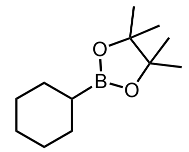

(7m)

^{1}H NMR (500 MHz, $CDCl_3$):

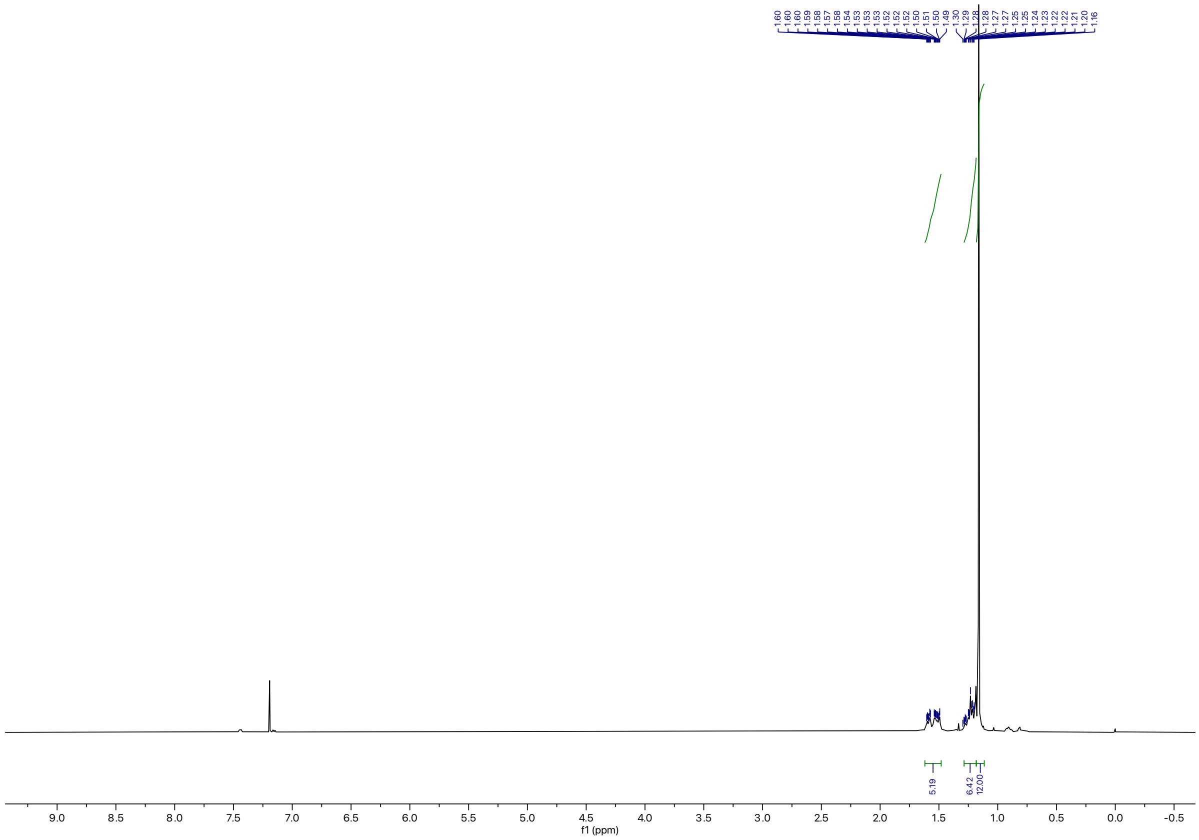

^{13}C NMR (126 MHz, $CDCl_3$):

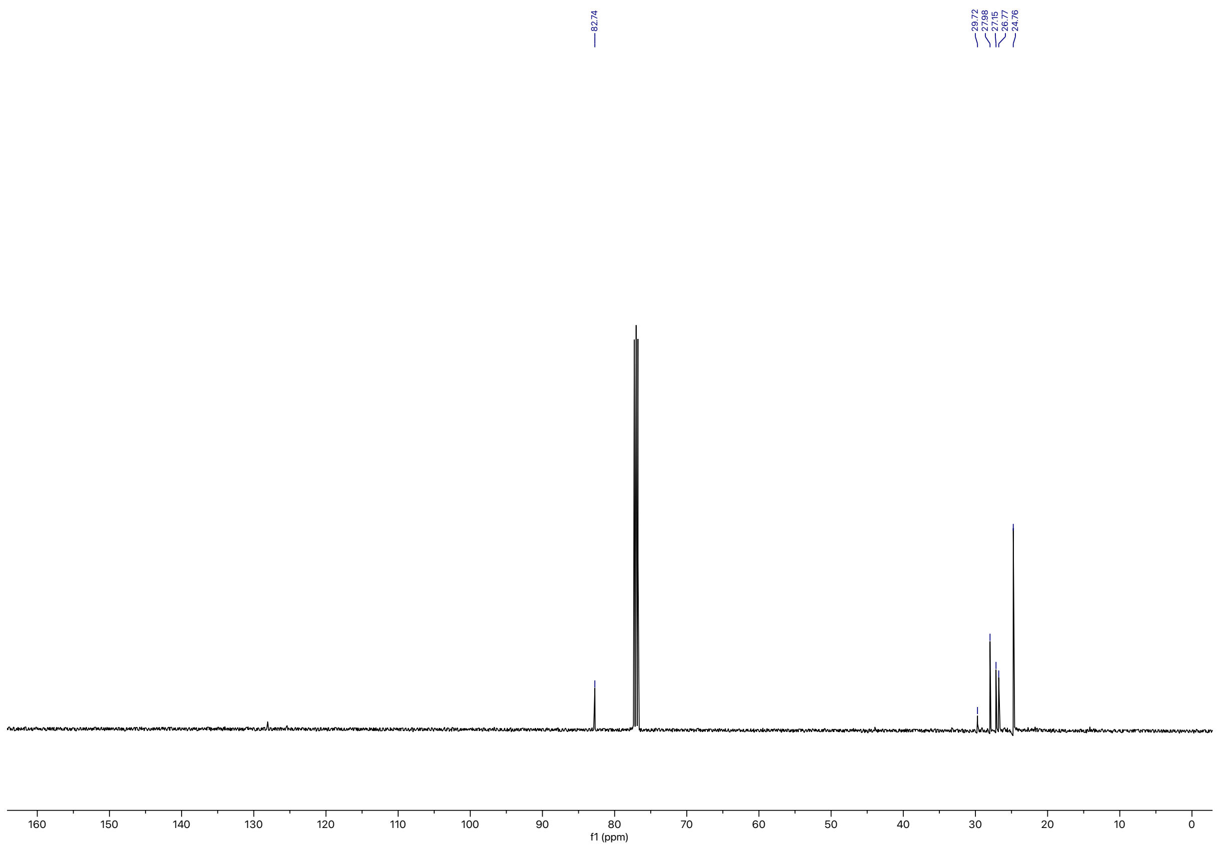

References:

(1) G. Zhang, J. Wu, S. Li, S. Cass and S. Zheng, *Org. lett.*, 2018, **20**, 7893-7897.
